# A virtual molecular tumor board to improve efficiency and scalability of delivering precision oncology to physicians and their patients

**DOI:** 10.1093/jamiaopen/ooz045

**Published:** 2019-10-07

**Authors:** Michael J Pishvaian, Edik M Blais, R Joseph Bender, Shruti Rao, Simina M Boca, Vincent Chung, Andrew E Hendifar, Sam Mikhail, Davendra P S Sohal, Paula R Pohlmann, Kathleen N Moore, Kai He, Bradley J Monk, Robert L Coleman, Thomas J Herzog, David D Halverson, Patricia DeArbeloa, Emanuel F Petricoin, Subha Madhavan

**Affiliations:** 1 Lombardi Comprehensive Cancer Center, Georgetown University Medical Center, Washington DC, USA; 2 Perthera, Inc, McLean, Virginia, USA; 3 Innovation Center for Biomedical Informatics, Georgetown University Medical Center, Washington DC, USA; 4 City of Hope Cancer Center, Duarte, California, USA; 5 Cedars-Sinai Medical Center, Los Angeles, California, USA; 6 Mark H. Zangmeister Cancer Center, Columbus, Ohio, USA; 7 Case Comprehensive Cancer Center, University Hospitals Seidman Cancer Center, Cleveland Clinic Taussig Cancer Institute, Cleveland, Ohio, USA; 8 Stephenson Oklahoma Cancer Center, University of Oklahoma, Oklahoma City, Oklahoma, USA; 9 Arizona Oncology, University of Arizona College of Medicine, Phoenix, Arizona, USA; 10 University of Texas, MD Anderson Cancer Center, Houston, Texas, USA; 11 University of Cincinnati Cancer Institute, University of Cincinnati, Cincinnati, Ohio, USA; 12 George Mason University, Fairfax, Virginia, USA

**Keywords:** precision informatics, precision oncology, virtual tumor boards, molecular tumor boards, implementation science

## Abstract

**Objectives:**

Scalable informatics solutions that provide molecularly tailored treatment recommendations to clinicians are needed to streamline precision oncology in care settings.

**Materials and Methods:**

We developed a cloud-based virtual molecular tumor board (VMTB) platform that included a knowledgebase, scoring model, rules engine, an asynchronous virtual chat room and a reporting tool that generated a treatment plan for each of the 1725 patients based on their molecular profile, previous treatment history, structured trial eligibility criteria, clinically relevant cancer gene-variant assertions, biomarker-treatment associations, and current treatment guidelines. The VMTB systematically allows clinician users to combine expert-curated data and structured data from clinical charts along with molecular testing data to develop consensus on treatments, especially those that require off-label and clinical trial considerations.

**Results:**

The VMTB was used as part of the cancer care process for a focused subset of 1725 patients referred by advocacy organizations wherein resultant personalized reports were successfully delivered to treating oncologists. Median turnaround time from data receipt to report delivery decreased from 14 days to 4 days over 4 years while the volume of cases increased nearly 2-fold each year. Using a novel scoring model for ranking therapy options, oncologists chose to implement the VMTB-derived therapies over others, except when pursuing immunotherapy options without molecular support.

**Discussion:**

VMTBs will play an increasingly critical role in precision oncology as the compendium of biomarkers and associated therapy options available to a patient continues to expand.

**Conclusion:**

Further development of such clinical augmentation tools that systematically combine patient-derived molecular data, real-world evidence from electronic health records and expert curated knowledgebases on biomarkers with computational tools for ranking best treatments can support care pathways at point of care.

## BACKGROUND AND SIGNIFICANCE

Precision oncology holds great promise but its implementation is time-consuming and requires the coalescence of patient-centric molecular and clinical data through user-friendly informatics tools.^1^ The increasing number of clinical laboratories offering comprehensive molecular profiling tests has further complicated the choices a physician must make in tailoring therapies based on molecular profiling results and evaluate the benefit of treatment options including off-label therapies or clinical trials associated with these results.^2^ Standard practice guidelines for medical oncologists often recommend that all patients with cancer be considered for a clinical trial; yet no guidelines exist for navigating the increasingly complex landscape of clinical trials and therapeutic agents applicable to each patient.

To address these challenges, molecular tumor boards that assess the clinical action ability of biomarkers are becoming critical. These tumor boards can help delineate individualized treatment strategies for patients, improve diagnosis and significantly increase the identification of eligible clinical trial options for patients in the current complex healthcare environment.^3^

Although many cancer centers have developed molecular tumor boards,^4^^,^^5^ convening busy medical oncologists and domain experts in a conference room to discuss the role of biomarkers in treatment selection is not always logistically feasible. This has an impact on the routine use of molecular reports in cancer care, which is perceived as a major barrier by patients.^6–9^ Since a majority of cancer patients are treated in community clinics with limited access to disease-specific molecular expertise,^10^ molecular tumor boards that allow relevant experts to review each case and make treatment recommendations by integrating molecular test results, data from electronic health records, knowledgebases with mechanistic evidence of biomarkers and current clinical guidelines will help democratize access to academic medical center-level care in the community. Making molecular tumor boards virtual will allow cancer care teams to communicate in a time and location independent manner, collaborate on decision making, share the latest research and discuss eligible clinical trials.^11^^,^^12^

## OBJECTIVES

In this study, we present an informatics platform that uses an innovative cloud-based virtual molecular tumor board (VMTB) technology that includes a knowledgebase, rules engine, scoring model, an asynchronous chat room, and a reporting tool to provide a collaborative environment for precision oncology-driven cancer care. This platform integrates different data types about a patient including past medical and treatment histories, pathology reports and genomic, proteomic and other molecular profiling results from testing laboratories, all designed to streamline the identification and prioritization of treatment options including on-label, clinical trial and in rare cases, and off-label options when clinically required. Subspecialty experts participating in the VMTB use this information to produce reports summarizing the rationale for the ranked list of personalized therapy options for each patient. The VMTB technology allows clinicians to communicate with each other asynchronously via a “chat room” interface module to discuss complex cases. Here, we describe the informatics workflow within VMTB and the clinical team’s experience in using the VMTB to generate treatment plans in 1725 cancer patients across US-based cancer clinics and health systems.

## MATERIALS AND METHODS

### Overview of the VMTB workflow

**Figure 1.**
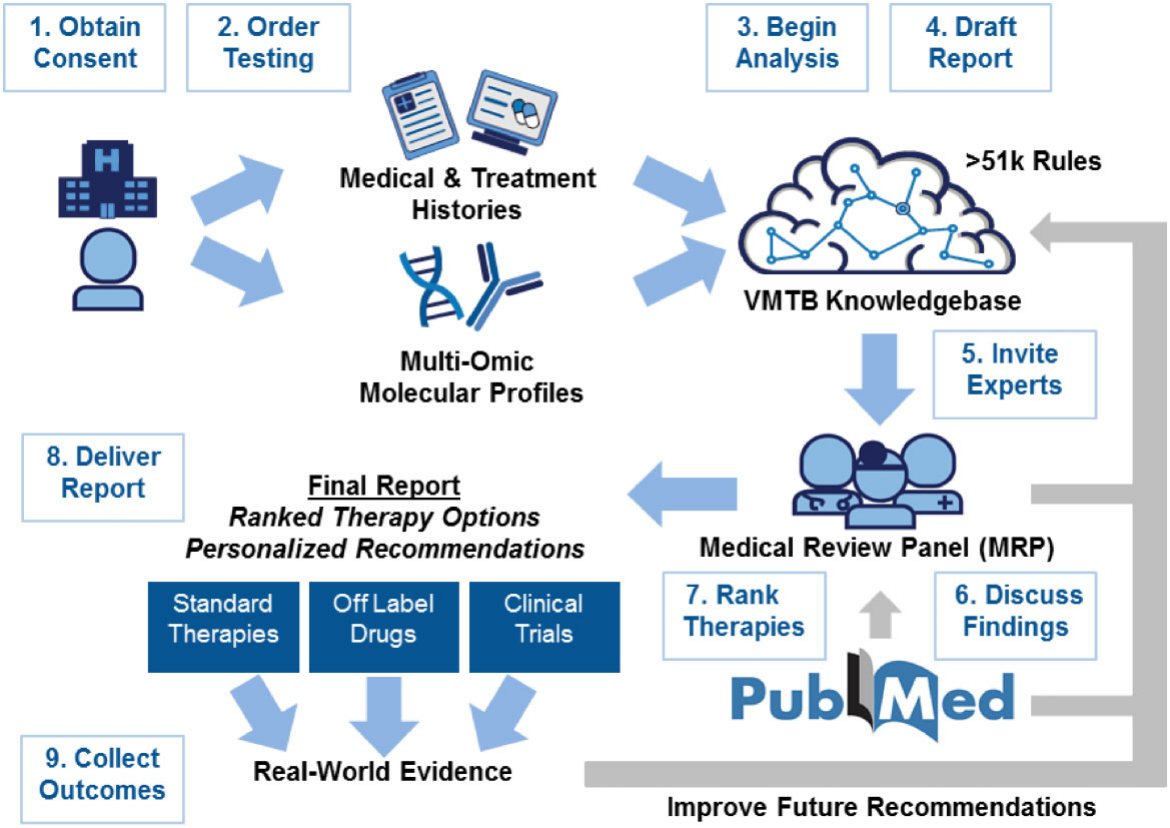
Overview of the virtual molecular tumor board workflow. Patients referred to Perthera’s virtual molecular tumor board (VMTB) for personalized treatment recommendations are consented to an IRB-approved registry. After obtaining medical records and facilitating the successful completion of molecular testing by commercial laboratories, structured data, and unstructured documents are integrated into a HIPAA-compliant cloud-based knowledgebase. A preliminary report is produced through our comprehensive, rules-based data engine which produces the initial ranked therapies. VMTB users are invited to review individual cases in a secure online portal that features an asynchronous chat window and the ability to formulate, modify, and rank personalized recommendations. After discussions are closed, a final report consisting of ranked therapy options consisting of on-label, off-label, and experimental interventions are delivered to patients and their treating oncologists. Workflow enhancements include automated treatment matching algorithms that provide a preliminary set of therapy options with clinical trial recommendations for VMTB users to discuss and modify. This iteratively improved platform aims to provide a patient-centered platform for scalable precision oncology that leverages real-world outcomes (additional consent provided for post-report records collection), curated literature evidence and domain-specific clinical expertise.

We have developed an informatics platform to systematically integrate patient-derived molecular data with clinical records to generate ranked lists of therapies and clinical trials for cancer patients (Figure 1). The platform compares patient data to a curated knowledgebase sourced from a variety of clinically relevant cancer gene variant databases—including COSMIC,^13^ mycancergenome,^14^ OncoKB,^15^ CIViC,^16^ clinical trials data from clinicaltrials.gov,^17^ and the European Union clinical trials register^18^—and drug–gene relationships from peer-reviewed journals and conference proceedings, which were curated based on published literature and continually updated based on clinician reports generated using the VMTB. A comprehensive rules engine, featuring over 10 databases and over 51 000 heuristic rules, determines appropriate therapies and clinical trials for each patient. The VMTB includes software tools such as Google Firebase, to facilitate a collaborative, asynchronous review by medical and scientific experts, as well as to support operational workflows such as sending notifications to relevant clinical staff when key steps have been completed. After review by the assigned clinician and approval by the invited medical review panel (MRP), including a quality assurance team, a final report is delivered via fax/email/mail to the patient’s treating oncologist—this is all facilitated by the VMTB. Patient outcomes were collected for a minimum of 6 months (up to 5 years) post report delivery.

### Focused cohort analysis: user experience in using the VMTB for advocacy organization referred patients

As a focused cohort analysis, we describe our experience in using our VMTB platform for a subset of our cancer patients from across the continental United States through referrals from advocacy groups (including the Pancreatic Cancer Action Network [PanCAN],^19^ Zero—The End of Prostate Cancer, Lung Cancer Alliance, and Hope for Stomach Cancer). The clinical teams, patients, testing laboratories, informatics, and operational teams were geographically dispersed and hence required a virtual platform to integrate data and develop consensus for each patient’s report through the VMTB. Of the 1725 cancer patients used for this analysis, 1163 patients were diagnosed with pancreatic adenocarcinoma, 103 with pancreaticobiliary tumors, 104 with other gastrointestinal (GI) cancers and 355 with non-GI cancers (77 breast, 55 ovarian, 51 sarcoma, 48 lung, 25 uterine, 30 prostate, and 58 other cancers). Molecular data used as input to the VMTB was obtained from commercial testing labs, primarily Foundation Medicine (Cambridge, MA) for genomics and either Caris Life Sciences (Irving, TX) or NeoGenomics (Fort Myers, FL) for IHC-based testing. Patients were consented to an IRB-approved registry protocol to obtain progress notes and other outcomes after delivery of final reports. Data transfer agreements were also established with various high-volume laboratories to enable the sharing of raw sequencing data and structured molecular profiling results via secure FTP servers in a machine-readable format. Laboratory-provided physician reports were also obtained as PDF files and made available in the VMTB user interface alongside any relevant pathology reports.

### Therapy option scoring and ranking

**Figure 2.**
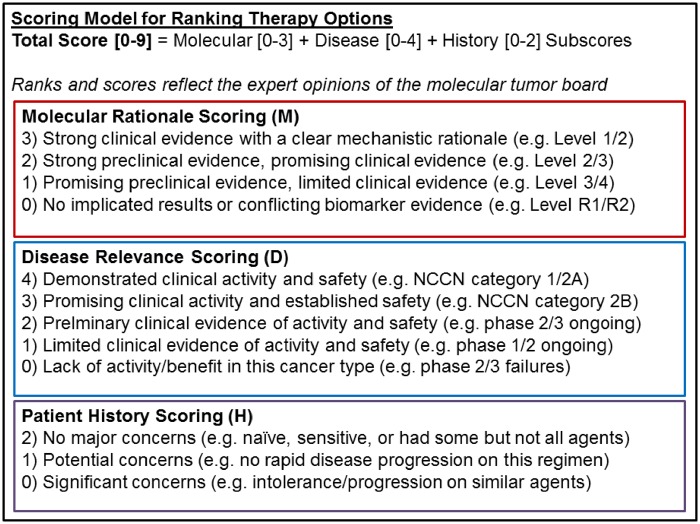
A scoring model designed for VMTB users to rank therapy options based on an individual patient’s multiomic molecular data, clinical information, and treatment history in accordance with current guidelines for biomarker associations and standard of care. A total score between 0 and 9 was determined for each therapy option by adding the subscores from three vectors corresponding to the predictive value of the molecular findings, the perceived clinical activity of the regimen in the specific cancer type and additional considerations regarding the patient’s prior treatment history. Unlike existing scoring models, the molecular vector was designed to reflect the expert opinions of the VMTB panel based on the patient’s entire multiomic profile that may include both positive predictors (eg, pAKT positive, PTEN loss) and negative predictors (eg, ERCC1 high) for a regimen that includes one or more therapeutic agents (eg, a PI3K inhibitor plus a platinum agent on a clinical trial).

We designed a scoring model suitable for prioritizing therapy options for each patient analyzed through VMTB (Figure 2). Each therapy option is ranked utilizing the rules based engine which was aligned to three subscoring vectors that reflect the molecular rationale, the disease relevance and the patient’s treatment history. The molecular rationale score used to summarize multiomic biomarker data from various testing laboratories extended best practices for assessing the therapeutic action ability of molecular alterations published by the ClinGen Somatic Cancer Working Group,^20^ Association for Molecular Pathology,^21^ ESCAT,^22^ and OncoKB.^15^ The strength of evidence supporting a therapy option based on the entire molecular profile of a patient was evaluated and distilled as either strong (3), moderate (2), weak (1), or neutral (0). Similar to other scoring models,^15^ the highest score of 3 is typically supported by both clinical evidence and a clear mechanistic understanding of the therapeutic association. FDA-approved companion diagnostic tests supported by prospective, randomized clinical studies represented the gold standard with the highest level of evidence in the model. An example of a molecular rationale of high strength is that for Olaparib, a PARP inhibitor indicated as second line maintenance treatment in advanced epithelial ovarian, fallopian tube, or primary peritoneal cancer for tumors with pathogenic or suspected pathogenic BRCA1 or BRCA2 mutations, both germline and somatic.

The disease relevance score accounted for the overall evidence of activity/safety in the patient’s cancer type and was scored separately from the molecular and history vectors. Drugs and combinations of drugs with a high level of evidence supporting efficacy and/or safety in particular disease types had the highest disease relevance score. Strong efficacy/safety data in another cancer type led to moderate disease scores, while a lack of efficacy and/or safety data in any cancer type led to low disease scores.

The patient’s medical history score was designed to account for previous exposure to one or more of the recommended agents (or similar classes of agents based on toxicity profiles and/or mechanisms of action). Drugs to which the patient has had no prior exposure received the highest score, while exposure (without disease progression) led to lower scores.

Based on the VMTB’s discussions during each case review, curators manually added, removed, or modified the evidence supporting the therapy options, which in turn updated the scoring system and therapy rankings.

### Automated clinical trial matching

**Figure 3.**
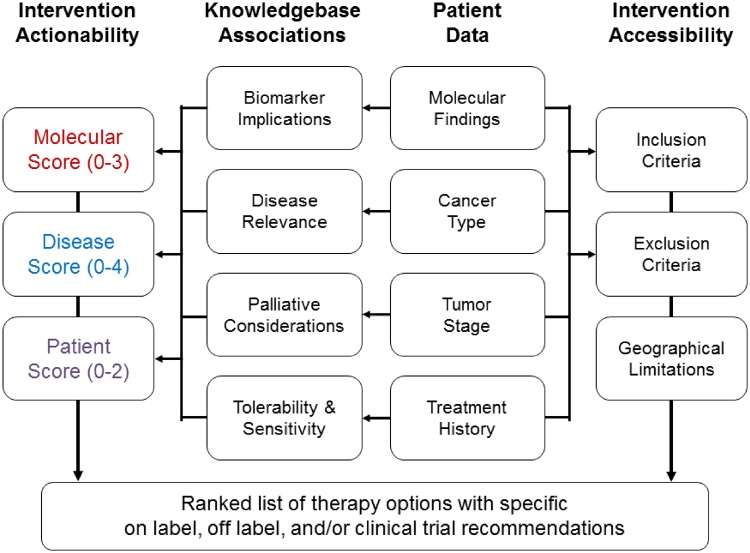
Workflow to generate a customized list of on-label, off-label, and/or clinical trial therapy options. Therapy options listed in reports finalized by the VMTB take into account both the actionability and accessibility of specific interventions available to a patient in clinical trials and as on or off-label treatments. We implemented a customized trial matching algorithm to identify relevant trials for an individual patient by aligning patient data to structured eligibility criteria. These data can also be used to provide VMTB users with a preranked list of trials by approximating the three therapy option scoring vectors (molecular rationale, disease relevance, and patient history).

To identify all appropriate, open clinical trials for each patient, we developed a computational framework that integrates and interprets the clinical characteristics of a patient’s tumor, the patient’s prior treatment history and molecular profiling data (Figure 3). For each clinical trial under consideration, the algorithm assigns a score to the therapeutic agents based on the scoring system described above. In addition to the three scoring vectors, the clinical trial matching algorithm also accounts for the city of residence of the patient. The patient data are matched to a curated database of annotated, structured eligibility criteria (eg, cancer type, tumor stage, line of therapy, biomarkers, etc.). The source of the clinical trial information comes from unstructured eligibility criteria found in public resources such as www.clinicaltrials.gov^17^ and European Union Clinical Trials Register.^18^

### Clinical trial search by medical oncologists

To assess the usefulness of VMTB in matching each patient’s molecular profile to appropriate trials, we asked a panel of 5 GI oncologists to assess the burden of finding appropriate clinical trials for patients with advanced pancreatic cancers. For this assessment, *each* clinician was presented with 3 hypothetical scenarios in which a patient with pancreatic cancer (1 neuroendocrine carcinoma, 2 adenocarcinomas) was being considered for a clinical trial after having received molecular profiling results. In each scenario, the oncologist was asked to identify the top 3 clinical trials for the patient using www.clinicaltrials.gov^17^ based on the genomic and proteomic findings in addition to cancer type, tumor stage, treatment history, age, sex, and geographical preferences. The amount of time spent searching for appropriate trials and researching relevant resources (eg, scientific literature on biomarkers and their associations with anticancer agents) was recorded. Trial ranks determined by the VMTB matching algorithm were compared to a score based on trial search conducted by oncologists (#1, #2, #3 rankings were assigned a value of 3, 2, or 1, respectively and added across all respondents for each scenario).

## RESULTS

As of December, 2018, the curated knowledgebase that supported the VMTB contained 51 165 heuristic rules. These rules captured relationships across 2064 clinical trials (arm-specific interventions structured with inclusion/exclusion eligibility criteria), 1015 therapeutic agents (chemotherapy, immunotherapy, targeted, and endocrine agents) and 195 biomarkers associated with sensitivity or resistance to therapies (4389 drug-gene mappings, 2133 distinct implicated variants, and 1461 curated therapy associations). Assertions in the knowledgebase were supported by 2731 scientific studies from peer-reviewed journals and conference proceedings.

**Figure 4.**
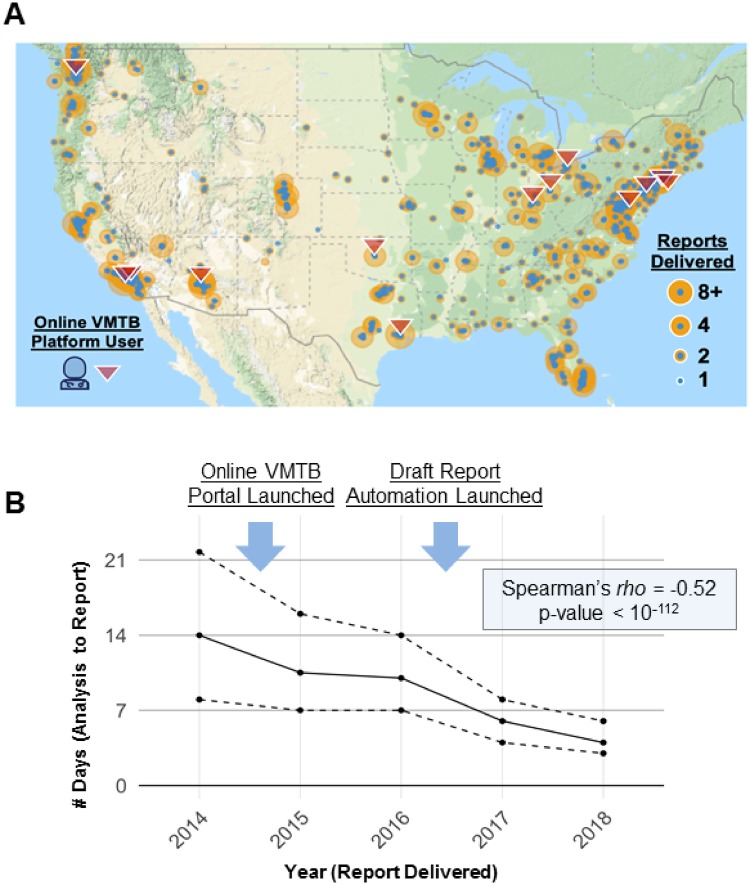
Overcoming geographical and logistical barriers using a scalable platform for precision oncology. (A) Geo map generated using Google Maps shows the spread of clinics where patients were seen for the cohort analysis presented in this study. Over 200 academic and community oncology practices (blue dots) were able to take advantage of the expertise of a small group of oncologists trained in precision oncology (red triangles) to deliver personalized treatment options to their patients. (B) After receiving molecular testing results and past medical and treatment history, turnaround time for case review by VMTB users decreased from a median of 14 days in 2014 to 4 days in 2018. Turnaround time was evaluated as the number of days between starting step 3 and completing step 8 as described in Figure 1. The solid line represents the median turnaround time and dashed lines represent 25th and 75th percentiles. A significant negative correlation (Spearman’s rho = −0.52; *P* = 2.9 × 10^−113^) was observed between the year of report delivery and the turnaround time.

Between 2013 and 2018, personalized reports were delivered across a wide geographic area (Figure 4A). Through the use of the VMTB over a 4-year period, the volume of cases reviewed in our advocacy organization cohort increased nearly 2-fold each year (46 in 2014, 188 in 2015, 354 in 2016, and 622 in 2017) while the medical review team members increased from 3 in 2014 to 5 in 2015, 10 in 2016, and 14 in 2017. Initially, cases were discussed via secure email by a MRP who iteratively formulated a report summarizing treatment options. With the first iteration of the VMTB software, users were able to log into a secure portal, discuss the case in a virtual chat room and modify the report contents using an online interface. In the second iteration, users were able to view documents related to the patient’s past medical history and laboratory testing results. In the third iteration, we streamlined case review for the VMTB using customized algorithms that integrate clinical and molecular data to generate a draft report with an initial set of ranked therapy options and corresponding clinical trial recommendations. Over time, the number of days necessary to review a case decreased substantially (*P* = 2.9 × 10^−113^ for the Spearman correlation test between the number of days from analysis to report and the year, downward trend also seen in Figure 4B). The VMTB databases (knowledgebase and patient databases) as well as the software application for online access were hosted on a HIPAA-compliant Amazon Web Services Cloud environment.

### Focused cohort study results: the VMTB platform integrates multiomic molecular data to provide matched therapy options

Treatment recommendations provided by molecular testing companies rarely account for information about the patient’s specific cancer, treatment history, and data from other testing laboratories. We systematically compared treatments listed in laboratory-provided reports to the ranked therapy options selected through VMTB review in a subset analysis (*N* = 642). By considering patients’ previous treatment histories, VMTB users avoided standard therapy options in 64% of patients who were either resistant to or had previously developed serious adverse events to one or more of the standard agents. Additionally, VMTB reviewers avoided recommending the off-label use of single agent MEK inhibitors, CDK4/6 inhibitors and mTOR inhibitors, in certain cancer types (despite the fact that these recommendations were routinely listed in the patient’s laboratory-provided reports), due to the lack of sufficient clinical evidence. Overall, the VMTB provided more biomarkers with therapy options compared to the commercial lab reports in 503 out of 642 patients (*P* = 1.5 × 10^−46^ for comparing this proportion to 50%), as well as more markers specifically with clinical trial options in 496 out of 625 patients (*P* = 1.6 × 10^−48^ for comparing this proportion to 50%; Table 1). Proteomic IHC profiling had an additional impact—protein results influenced on-label treatment recommendations in 229 patients (36%) and off-label treatment recommendations in 80 (12%) patients.

**Table 1.** Comparison of the number of patients having more markers with therapy options from the VMTB report versus the commercial lab test report

Therapy recommendations	# of patients			Test of proportions: Null hypothesis is that percentage of patients with more markers from VMTB report = 50%
	More markers from VMTB report (percentage)	More markers from commercial lab test report (percentage)	Total assigned to specific therapy recommendations	
Off-label	171	50	221	6.9 × 10^−16^
	(77%)	(23%)		
Clinical trial	496	129	625	1.6 × 10^−48^
	(79%)	(21%)		
Any therapy	503	139	642	1.5× 10^−46^
(On-label/Off-label/Clinical trial)	(78%)	(22%)		

*Note:* The number of patients having more markers with therapy options from the VMTB report, compared to the commercial lab test report for either off-label, clinical trial, or any therapy option. Note that the total number of patients within this cohort considered is 642, out of which all have at least one marker with a therapy recommendation, but only 221 have markers with off-label indications and 625 have markers with clinical trials indications. The rightmost column gives the p-value from a test of proportions comparing the fraction of patients with more markers from the VMTB report (vs the commercial lab test report) to 0.5, using the prop.test function in the *R* statistical programming language.

### Clinical trial search results by clinicians lack consensus

**Figure 5.**
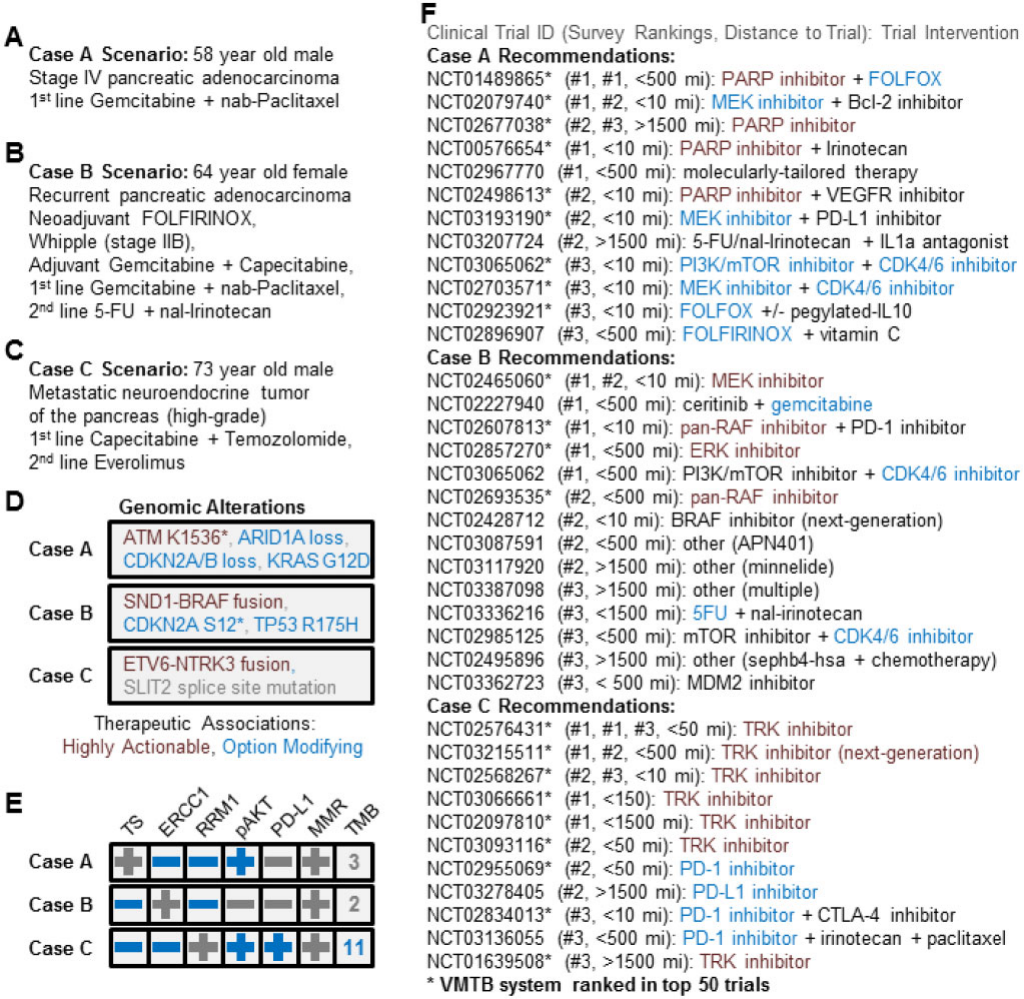
The VMTB system prioritizes clinical trials that are consistent with those suggested by medical oncologists. Clinical trial search results from 5 clinicians asked to independently recommend the top 3 clinical trials for 3 mock pancreatic cancer cases by searching www.clinicaltrials.gov. Case-specific rankings generated by the VMTB matching algorithm correlated significantly with consensus-based rankings of trial search responses (Spearman’s rho = 0.36, *P*-value = 0.027) with a high degree of sensitivity (57% of oncologists who participated in the trial search recommended trials prioritized in the top 50 by the VMTB system). (A–E) show the case descriptions for the three cases selected; *F* shows the trial search results for each case and their rankings by oncologists.

We presented 3 hypothetical pancreatic cancer cases (Figure 5A–C) to 5 medical oncologists with expertise in GI cancers and recorded each clinician’s top 3 clinical trial suggestions for each patient. The time spent identifying sets of 3 trials was 20 minutes on average across all 15 trial search responses with some variation between doctors (10–30 minute averages) and between case scenarios based on the complexity of the case (15–30 minute averages). This suggests that an automated system such as the VMTB may help standardize trial selection by multiple oncologists in a single practice.

The clinical trials search presented to oncologists was designed so that for each patient, only one biomarker was linked to a therapy with the highest level of actionability (Figure 5D) based on our scoring model in the VMTB: an *ATM* mutation in case A, a *BRAF* fusion in case B and an *NTRK3* fusion in case C (Of note: This clinical trial search assessment was performed prior to the FDA approval of larotrectinib for patients with tumors harboring NTRK fusions). However, 4 of 5 oncologists chose a therapy targeting the *ATM* mutation and only 3 of 5 respondents chose a therapy targeting the *BRAF* fusion. We note that for case A, one oncologist listed a trial evaluating physician’s discretion versus molecularly tailored therapy (NCT02967770) as the top recommendation; we assume that this was intended to target the *ATM* mutation. All oncologists chose a Trk inhibitor as the top trial for the patient with an *NTRK3* fusion.

Geographically, the trials selected by the five oncologists varied widely by distance from the patients. Of the four PARP inhibitor trials recommended for case A, only two of them were within a 2-hour drive of the patient’s city of residence. Similarly, of the four trials targeting the *BRAF* fusion in case B, only two were within a 2-hour drive. This suggests that automated tools in the VMTB can further inform other variables such as patient’s preferred location.

### Real-world outcomes suggest physicians preferentially treated their patients in accordance with VMTB recommendations

**Figure 6.**
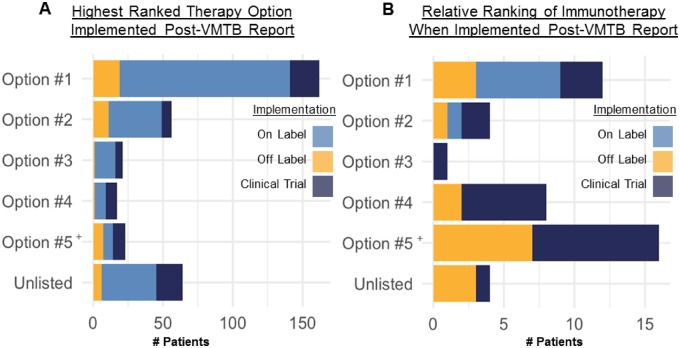
Treating physicians preferentially implemented top ranked therapy options listed on VMTB reports. (A) Distribution of the highest ranked therapy option chosen by each patient and their treating oncologist (1 per patient, lesser ranked implementations omitted). (B) Distribution of therapy option rankings implemented that included an immune checkpoint inhibitor (PD-1/PD-L1/CTLA-4/OX40 antibody). It is important to note that immunotherapy was nearly universally recommended (with or without molecular support) but primarily in the context of a clinical trial and rarely as an off-label option.

Treating oncologists frequently implemented VMTB report-listed therapies (Figure 6A). We analyzed real-world outcomes from a cohort of 343 patients who initiated a new therapy post-VMTB report and found that 279 (81%) patients received at least 1 therapy that was consistent with a report-listed option. The remaining 64 patients were primarily put on chemotherapy regimens (*n* = 40) and nonbiomarker-driven clinical trials (*n* = 15) which were reasonable choices in those clinical contexts. It is important to note that VMTB reviewers were not responsible for treating the patient, but could still track the implementation of non-VMTB-listed therapies via progress notes from the treating oncologist.

When physicians implemented a VMTB report-listed therapy, we found that patients frequently received highly ranked therapy options (Figure 6A). Of the 343 patients who initiated a new therapy postreport, 47% received a top scoring option. Cumulatively, 64%, 70%, 75% of patients received at least one of the top 2, 3, or 4 scoring options, respectively. Standard therapies generally received higher scores than nonstandard therapies except in the presence of highly actionable molecular findings or when standard options had already been exhausted.

When a patient initiated a new treatment post-report that included an immuno-oncology agent (*n* = 45), we found no correlation between the rank of the VMTB-listed immunotherapy option and its relative rank compared to other options provided to the patient’s treating oncologist (Figure 6B). In these 45 VMTB reports, immunotherapy was listed within the top 3 scoring options in only 17 reports (38%). For the 28 (62%) patients who received an immunotherapy that was either ranked lower than the top 3 scoring options or not explicitly recommended at all, we observed that 9 of these 28 patients did in fact receive at least one of the top 3 report-listed recommendations before or after receiving an immunotherapy.

### Implementation of actionable therapies is driven by clinical trial accessibility

Although the best management for patients with many advanced cancers is in a clinical trial according to the NCCN guidelines,^23^ patients often lack access to open study sites despite the actionability of their molecular profiling results. The VMTB integrates geographical information such as the mileage between the patient and the nearest actively recruiting facility for each recommended clinical trial. We analyzed previous cases that received VMTB reports and identified 10 patients who received a PARP inhibitor either on a clinical trial (*n* = 4) or off-label (*n* = 6). For patients who received a PARP inhibitor on a clinical trial, the nearest recruiting site was located within 150 miles for 3 out of 4 patients. For the 6 patients who received off-label PARP inhibitors, no immediately accessible PARP inhibitor trials were recommended within 150 miles.

### Increased enrollment in clinical trials for VMTB-moderated patients

Historically, less than 5% of pancreatic cancer patients enroll in clinical trials.^24^^,^^25^ However, we found that among all outcomes tracked patients who initiated a new therapy after receiving a report, 16% implemented an off-label therapy and 22% enrolled in a clinical trial (*P* = 7.2 × 10^−34^ for comparing this proportion of 56/259 patients to 5%, which represents the upper bound of pancreatic cancer patients who enroll in trials, using prop.test in *R*). These findings suggest that the options presented in the VMTB reports could influence therapeutic decisions; however, we acknowledge that this was a highly selected population of patients that were also highly motivated among other confounding factors. Nevertheless, we anticipate that molecularly tailored selection of clinical trials will be an integral approach that promotes clinical trial enrollment and improves patient outcomes, as has been demonstrated in previous biomarker-driven studies.^26^ Data to generate figures 4B, 6A and 6B are available in dryad: Michael J Pishvaian, E.M.B., R Joseph Bender, Shruti Rao, Simina M Boca, Vincent Chung, Andrew E Hendifar, Sam Mikhail, Davendra PS Sohal, Paula R Pohlmann, Kathleen N Moore, Kai He, Bradley J Monk, Robert L Coleman, Thomas J Herzog, David D Halverson, Patricia DeArbeloa, Emanuel F Petricoin III, Subha Madhavan (2019), Data from: A virtual molecular tumor board to improve efficiency and scalability of delivering precision oncology to physicians and their patients. doi:10.5061/dryad.bs0p30k.

## DISCUSSION

As comprehensive molecular profiling tests expand beyond genomics only, to include proteomics, phosphoproteomics, metabolomics, and future molecular analyses, the complexity of the input data and treatment options available to a patient are exploding. The medical oncology ecosystem operates in an overburdened environment and the ability of oncologists to keep up with the ever-expanding lists of biomarkers, treatment matching rules, cancer biology, single, and combination therapies will become untenable. This necessitates computationally driven clinical augmentation tools with a human-in-the-loop framework to ensure accurate and high quality treatment matching, that clinicians, molecular labs or computational systems alone cannot provide.

Here, we have presented the development, real-world implementation and testing of a novel VMTB technology that enables geographically dispersed molecular tumor board members to virtually assess and discuss the clinical actionability of molecular markers found in each patient and develop consensus and ranking for best treatment options. Such a platform allows for implementing a VMTB to patients anywhere in a geographically unbound and time-independent manner. The VMTB system also relies on human efforts from patient coordinators, tissue coordinators, software developers, technical support staff, computational biologists, scientific experts, and quality assurance reviewers to collect and prepare the datasets for review by the clinical team. While the VMTB analysis described herein was underpinned by a focused subset of patients referred to us by cancer advocacy organizations such as the PanCAN’s Know Your Tumor Program,^27^ the platform and the scoring model presented here are entirely generalizable to any cancer care ecosystem. This is a critical aspect of making tumor board software frameworks successful so that oncologists with diverse backgrounds can take advantage of it.

We also recognized that too many levels in the scoring models were confusing to VMTB users and revised the scoring model to reflect the Association of Molecular Pathology’s tiers of evidence for somatic variations (Supplementary Figure S1**).**^21^ Prospective approaches to precision oncology have focused on recommending relevant clinical trials to patients based on their molecular profile.^28^ Despite the presence of a potentially targetable alteration, actionability does not guarantee accessibility of the suggested intervention. One recent study found that 19 out of 95 (20%) patients were unable to enroll in a recommended study due to trial eligibility restrictions or inconvenient travel distances,^28^ highlighting the barriers to the delivery of patient care. Off-label therapies are more geographically accessible than clinical trials but may be inaccessible for reimbursement or other reasons. Similarly, obtaining investigational agents under compassionate use can be time-consuming and complicated from a regulatory standpoint.^29^ Based on the continually expanding availability of new FDA-approved drugs, clinical trials, and molecular profiling panels, we anticipate that oncologists will encounter an overwhelming number of therapy options moving forward, emphasizing the need for innovative solutions that foster collaborative and efficient treatment planning. Furthermore, human oversight will be critical to the adoption of novel data-driven technologies (eg, artificial intelligence, machine learning, and recommender algorithms^30^) in cancer care.

We anticipate that this cloud-based technology for implementing precision oncology could be extended to facilitate collaborative diagnosis and treatment planning between dispersed networks of medical experts using molecular data to care for patients at academic and community centers alike across any disease type and use cases, including for pharmacogenomics and rare genetic disease diagnosis use.

## CONCLUSION

While significant challenges remain in demonstrating the benefit, safety, and cost-effectiveness of comprehensive molecular profiling in routine cancer care, a multitude of efforts are underway including umbrella/basket trials (eg, TAPUR,^31^ MATCH^32^), initiatives that capture outcomes from targeted therapy decisions (eg, ClinGen,^33^ GA4GH^34^), data standards and interoperability tools (eg, SMART Cancer Navigator^35^), as well as studies by individual molecular tumor boards.^4^^,^^5^^,^^8^^,^^9^^,^^11^^,^^12^^,^^28^ Lab-agnostic data models and software frameworks are needed to share data on rare tumors across institutions, such as those being developed by the Global Alliance for Genomic Health (GA4GH)’s Variant Interpretation for Cancer Consortium (VICC). Support and resources are needed for expert curation of clinically relevant cancer variant curation and computable interfaces such as in CIViC^16^ and ClinVar.^36^ We also recognize that the scoring models for ranking cancer treatments will evolve with new discoveries of biomarkers and treatments, especially those involving combinations of immuno-oncology, targeted and chemotherapeutic agents and the need to address emerging evidence for markers of possible resistance that are context-dependent (eg, STK11 mutations for PD-1 inhibitors in KRAS mutant lung cancers.^37^ Furthermore, rules based data engines and scoring paradigms will become increasingly complex with novel combinations aimed at overcoming resistance.^38^

Through continued development of scalable VMTB systems that are agnostic to individual hospital systems, molecular testing laboratories, or pharmaceutical companies; clinicians can more freely and efficiently implement outcomes-based medicine, encounter a wider diversity of cases to train the next generation of clinicians and clinical informaticians and harness the biomarker-specific and disease-specific expertise of fellow users.

## FUNDING

This work was partly funded by the Lombardi cancer center support grant (NCI P30 CA51008), the Georgetown-Howard Universities CTSA (NCATS TR000102), grant NIH-NCI R21 CA220390 and the Pancreatic Cancer Action Network’s Know Your Tumor Program.

## AUTHOR CONTRIBUTIONS

MJP, EMB, SM designed the study. MJP, EMB, SM, SR, SB contributed to writing the manuscript. SB conducted statistical analysis shown in Figure 4B. RJB, VC, AEH, SM, DPSS, PRP, KNM, KH, BJM, RLC, TJH, DDH, PD, and EFP helped design, conduct the study and review the manuscript.

## COMPETING INTERESTS

EMB, RJB, DDH, and PD are/were employed by Perthera, Inc (McLean, VA, USA). EFP III is co-founder of and consultant to Perthera Inc. MJP, EFP III and SM are/were on Perthera, Inc’s scientific advisory board. Perthera is a for-profit healthcare company. Perthera is not a laboratory and does not receive financial compensation in return for facilitating the ordering of molecular profiling from external testing laboratories. 

## Supplementary Material

ooz045_Supplementary_Data

## References

[1] Rieke DT, Lamping M, Schuh M, et al Comparison of Treatment Recommendations by Molecular Tumor Boards Worldwide. JCO Precis Oncol 2018; (2): 1–14. 10.1200/PO.18.00098 35135153

[2] Kurzrock R, Colevas AD, Olszanski A, et al NCCN Oncology Research Program's Investigator Steering Committee and NCCN Best Practices Committee molecular profiling surveys. J Natl Compr Canc Netw 2015; 13 (11): 1337–46. 26553764 10.6004/jnccn.2015.0163

[3] Charara RN, Kreidieh FY, Farhat RA, et al Practice and impact of multidisciplinary tumor boards on patient management: a prospective study. J Glob Oncol 2017; 3 (3): 242–9. 28717766 10.1200/JGO.2016.004960 PMC5493220

[4] Harada S, Arend R, Dai Q, et al Implementation and utilization of the molecular tumor board to guide precision medicine. Oncotarget 2017; 8 (34): 57845–54. 28915716 10.18632/oncotarget.18471 PMC5593688

[5] Marshall CL, Petersen NJ, Naik AD, et al Implementation of a regional virtual tumor board: a prospective study evaluating feasibility and provider acceptance. Telemed J E Health 2014; 20 (8): 705–11. 24845366 10.1089/tmj.2013.0320 PMC4106373

[6] Patel M, Kato SM, Kurzrock R. Molecular tumor boards: realizing precision oncology therapy. Clin Pharmacol Ther 2018; 103 (2): 206–9. 29134641 10.1002/cpt.920 PMC5760337

[7] Perera-Bel J, Hutter B, Heining C, et al From somatic variants towards precision oncology: evidence-driven reporting of treatment options in molecular tumor boards. Genome Med 2018; 10 (1): 18. 29544535 10.1186/s13073-018-0529-2 PMC5856211

[8] Basse C, Morel C, Alt M, et al Relevance of a molecular tumour board (MTB) for patients' enrolment in clinical trials: experience of the Institut Curie. ESMO Open 2018; 3 (3): e000339. 29636991 10.1136/esmoopen-2018-000339 PMC5890857

[9] Tafe LJ, Gorlov IP, de Abreu FB, et al Implementation of a molecular tumor board: the impact on treatment decisions for 35 patients evaluated at Dartmouth-Hitchcock Medical Center. Oncologist 2015; 20 (9): 1011–8. 26205736 10.1634/theoncologist.2015-0097 PMC4571816

[10] O'Grady MA, Slater E, Sigurdson ER, et al Assessing compliance with national comprehensive cancer network guidelines for elderly patients with stage III colon cancer: the Fox Chase Cancer Center Partners' initiative. Clin Colorectal Cancer 2011; 10 (2): 113–6. 21859563 10.1016/j.clcc.2011.03.007 PMC3388796

[11] Knepper TC, Bell GC, Hicks JK, et al Key lessons learned from Moffitt's molecular tumor board: The Clinical Genomics Action Committee experience. Oncologist 2017; 22 (2): 144–51. 28179575 10.1634/theoncologist.2016-0195 PMC5330702

[12] Shea CM, Haynes-Maslow L, McIntyre M, et al Assessing the feasibility of a virtual tumor board program: a case study. J Healthc Manag 2014; 59 (3): 177–93. 24988672 PMC4116610

[13] Tate JG, Bamford S, Jubb HC, et al COSMIC: the catalogue of somatic mutations in cancer. Nucleic Acids Res 2019; 47 (D1): D941–D947. 30371878 10.1093/nar/gky1015 PMC6323903

[14] Taylor AD, Micheel CM, Anderson IA, et al The path(way) less traveled: a pathway-oriented approach to providing information about precision cancer medicine on my cancer genome. Transl Oncol 2016; 9 (2): 163–5. 27084433 10.1016/j.tranon.2016.03.001 PMC4833964

[15] Chakravarty D, Gao J, Phillips SM, et al OncoKB: a precision oncology knowledge base. JCO Precis Oncol 2017; 2017. 10.1200/PO.17.00011 PMC5586540 28890946

[16] Griffith M, Spies NC, Krysiak K, et al CIViC is a community knowledgebase for expert crowdsourcing the clinical interpretation of variants in cancer. Nat Genet 2017; 49 (2): 170–4. 28138153 10.1038/ng.3774 PMC5367263

[17] NIH: U.S. National Library of Medicine - ClinicalTrials.gov. https://clinicaltrials.gov/.

[18] EU Clinical Trials Register. https://www.clinicaltrialsregister.eu/.

[19] Fleshman J. Pancreatic cancer action network: advance research, support patients, and create hope. J Oncol Pract 2009; 5 (2): 98. 29443615 10.1200/JOP.0924502 PMC2790643

[20] Ritter DI, Roychowdhury S, Roy A, et al Somatic cancer variant curation and harmonization through consensus minimum variant level data. Genome Med 2016; 8 (1): 117. 27814769 10.1186/s13073-016-0367-z PMC5095986

[21] Li MM, Datto M, Duncavage EJ, et al Standards and guidelines for the interpretation and reporting of sequence variants in cancer: a joint consensus recommendation of the association for molecular pathology, American Society of Clinical Oncology, and College of American Pathologists. J Mol Diagn 2017; 19 (1): 4–23. 27993330 10.1016/j.jmoldx.2016.10.002 PMC5707196

[22] Mateo J, Chakravarty D, Dienstmann R, et al A framework to rank genomic alterations as targets for cancer precision medicine: the ESMO Scale for Clinical Actionability of molecular Targets (ESCAT). Ann Oncol 2018; 29 (9): 1895–902. 30137196 10.1093/annonc/mdy263 PMC6158764

[23] Winn RJ, Botnick W, Dozier N. The NCCN guidelines development program. Oncology (Williston Park) 1996; 10 (11 suppl): 23–8. 8953592

[24] Hoos WA, James PM, Rahib L, et al Pancreatic cancer clinical trials and accrual in the United States. J Clin Oncol 2013; 31 (27): 3432–8. 23960185 10.1200/JCO.2013.49.4823

[25] Matrisian LM, Berlin JD. The past, present, and future of pancreatic cancer clinical trials. Am Soc Clin Oncol Educ Book 2016; 35: e205–15. 27249725 10.1200/EDBK_159117

[26] Schwaederle M, Zhao M, Lee JJ, et al Impact of precision medicine in diverse cancers: a meta-analysis of phase II clinical trials. J Clin Oncol 2015; 33 (32): 3817–25. 26304871 10.1200/JCO.2015.61.5997 PMC4737863

[27] Pishvaian MJ, Bender RJ, Halverson D, et al Molecular profiling of patients with pancreatic cancer: initial results from the know your tumor initiative. Clin Cancer Res 2018; 24 (24): 6612–5027. 29954777 10.1158/1078-0432.CCR-18-0531

[28] Dalton WB, Forde PM, Kang H, et al Personalized medicine in the oncology clinic: implementation and outcomes of the Johns Hopkins molecular tumor board. JCO Precis Oncol 2017; 2017. 10.1200/PO.16.00046 PMC6039131 30003184

[29] Darrow JJ, Sarpatwari A, Avorn J, et al Practical, legal, and ethical issues in expanded access to investigational drugs. N Engl J Med 2015; 372 (3): 279–86. 25587952 10.1056/NEJMhle1409465

[30] Chen JH, Podchiyska T, Altman RB. OrderRex: clinical order decision support and outcome predictions by data-mining electronic medical records. J Am Med Inform Assoc 2016; 23 (2): 339–48. 26198303 10.1093/jamia/ocv091 PMC5009921

[31] Mangat PK, Halabi S, Bruinooge SS, et al Rationale and design of the Targeted Agent and Profiling Utilization Registry (TAPUR) study. JCO Precis Oncol 2018; 2018. 10.1200/PO.18.00122 PMC6312096 30603737

[32] Conley BA, Doroshow JH. Molecular analysis for therapy choice: NCI MATCH. Semin Oncol 2014; 41 (3): 297–9. 25023344 10.1053/j.seminoncol.2014.05.002

[33] Madhavan S, Ritter D, Micheel C, et al ClinGen Cancer Somatic Working Group - standardizing and democratizing access to cancer molecular diagnostic data to drive translational research. Pac Symp Biocomput 2018; 23: 247–58. 29218886 PMC5728662

[34] Lawler M, Siu LL, Rehm HL, et al All the world's a stage: facilitating discovery science and improved cancer care through the global alliance for genomics and health. Cancer Discov 2015; 5 (11): 1133–6. 26526696 10.1158/2159-8290.CD-15-0821

[35] Warner JL, Prasad I, Bennett M, et al SMART Cancer Navigator: a framework for implementing ASCO workshop recommendations to enable precision cancer medicine. JCO Precis Oncol 2018; 2018: 1–14. 10.1200/PO.17.00292 PMC6141039 30238071

[36] Landrum MJ, Lee JM, Benson M, et al ClinVar: improving access to variant interpretations and supporting evidence. Nucleic Acids Res 2018; 46 (D1): D1062–D1067. 29165669 10.1093/nar/gkx1153 PMC5753237

[37] Skoulidis F, Goldberg ME, Greenawalt DM, et al STK11/LKB1 mutations and PD-1 inhibitor resistance in KRAS-mutant lung adenocarcinoma. Cancer Discov 2018; 8 (7): 822–35. 29773717 10.1158/2159-8290.CD-18-0099 PMC6030433

[38] Kitajima S, Ivanova E, Guo S, et al Suppression of STING associated with LKB1 loss in KRAS-driven lung cancer. Cancer Discov 2019; 9 (1): 34–45. 30297358 10.1158/2159-8290.CD-18-0689 PMC6328329

