# Supplementary material for: A virtual molecular tumor board to improve efficiency and scalability of delivering precision oncology to physicians and their patients
Source: JAMIA Open. 2019 Oct 7;2(4):505–15. doi: 10.1093/jamiaopen/ooz045 (PMC6994017; doi:10.1093/jamiaopen/ooz045)
Supplement: ooz045_Supplementary_Data [file ooz045_supplementary_data.docx]

**A virtual molecular tumor board platform to improve efficiency and scalability of delivering precision oncology to physicians and their patients**

Michael J. Pishvaian, Edik M. Blais*, et al.

## Supplemental Figures


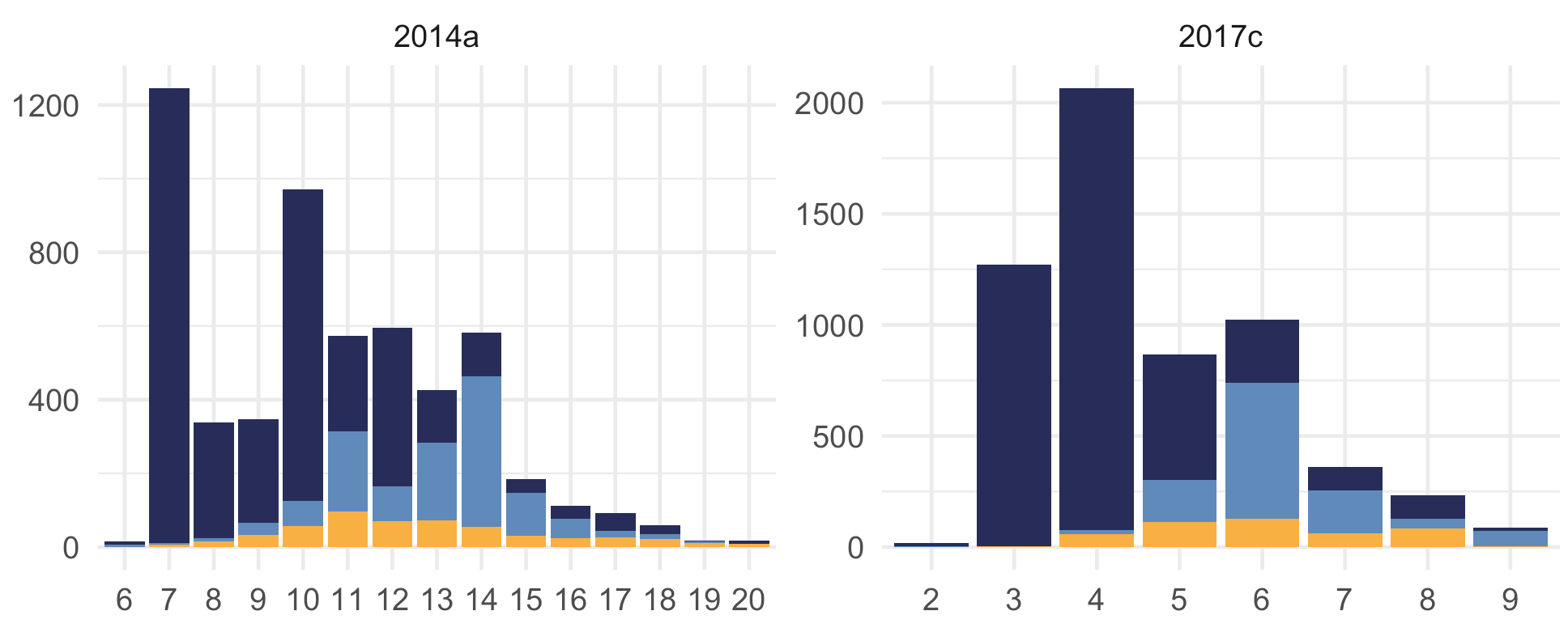


**Supplemental Figure S1** – Distributions of therapy option scores listed in personalized reports based on a current and previous scoring model. We found that too much versatility made it difficult for individual VMTB users to use each scoring value consistently over time and for multiple VMTB users to independently assign similar scores for similar therapies across patients with similar molecular/clinical/treatment backgrounds. All therapy option scores presented in this study were converted from the 2014a scoring model to the 2017c scoring model using the rationale established by VMTB users when designing the simplified scoring model.
